# A Call to Organize a Society

**Published:** 1865-08

**Authors:** M. DeCamp, A. W. Maxwell


### A CALL TO ORGANIZE A SOCIETY.

The few dentists representing the central portion of the State of Ohio in the American Dental Convention at White Sulphur Springs, held a meeting in the chapel on Wednesday evening, Aug. 2d, for the purpose of taking initiatory measures for effecting the organization of an association for the benefit of the members of the profession in that section of the State.

Dr. M. DeCamp, of Mansfield, was called to the chair, and Dr. A. W. Maxwell, of Galion, was appointed secretary. The chairman stated the object of the meeting, and remarks encouraging the project were made by Drs. H. E. Peebles, St. Louis, Mo., T. L. Buckingham, Philadelphia, Pa., C. Palmer, Warren, O., M. DeCamp, Wm. E. Dunn, Delaware, O., C. M. Kelsey, H. M. Edson, Mt. Vernon, O., J. B. Beauman, Columbus, O., and J. Taft, Cincinnati, O.

Dr. Maxwell offered the following resolution, which was adopted by the meeting.

*Resolved*, That we as dentists, representing the central portion of the State of Ohio, feeling the necessity for associated effort in promoting the interests of the profession in the above named territory, and believing that an organization may be effected which will accomplish good results in that direction, do agree to meet in the city of Mansfield, on Tuesday, the 5th day of September next, and that an invitation be extended to all dentists in the part of the State named, to meet with us, for the purpose of organizing a Dental Association.

Signed—M. DeCamp, J. B. Beauman, Wm. E. Dunn, C. M. Kelsey, H. M. Edson, C. Palmer, and A. W. Maxwell.

On motion of Dr. Kelsey, the chairman and secretary of this meeting were appointed a committee to draft a constitution and by-laws, to be presented to the meeting at Mansfield, September 5th. And on motion of Dr. Beauman, the same committee be authorized to have a printed notice of the meeting at Mansfield sent to each dentist in the central portion of the State.

M. DeCamp, *Pres.*
A. W. Maxwell, *Sec'y.*

